# Proteomic Insights into Bacterial Responses to Antibiotics: A Narrative Review

**DOI:** 10.3390/ijms26157255

**Published:** 2025-07-27

**Authors:** Sara Elsa Aita, Maria Vittoria Ristori, Antonio Cristiano, Tiziana Marfoli, Marina De Cesaris, Vincenzo La Vaccara, Roberto Cammarata, Damiano Caputo, Silvia Spoto, Silvia Angeletti

**Affiliations:** 1 Operative Research Unit of General Surgery, Fondazione Policlinico Universitario Campus Bio-Medico, 00128 Rome, Italy; s.aita@policlinicocampus.it (S.E.A.); v.lavaccara@policlinicocampus.it (V.L.V.); roberto.cammarata@policlinicocampus.it (R.C.); d.caputo@policlinicocampus.it (D.C.); 2 Operative Research Unit of Laboratory, Fondazione Policlinico Universitario Campus Bio-Medico, 00128 Rome, Italy; a.cristiano@policlinicocampus.it (A.C.); t.marfoli@policlinicocampus.it (T.M.); m.decesaris@policlinicocampus.it (M.D.C.); s.angeletti@policlinicocampus.it (S.A.); 3 Research Unit of Clinical Laboratory Science, Department of Medicine and Surgery, Università Campus Bio-Medico di Roma, Via Alvaro del Portillo, 21, 00128 Rome, Italy; 4 Department of Biomedicine and Prevention, PhD Program in Immunology, Molecular Medicine and Applied Biotechnologies, Tor Vergata University, 00133 Rome, Italy; 5 Research Unit of General Surgery, Università Campus Bio-Medico di Roma, 00128 Rome, Italy; 6 Diagnostic and Therapeutic Medicine Department, Fondazione Policlinico Universitario Campus Bio-Medico, 00128 Rome, Italy; s.spoto@policlinicocampus.it

**Keywords:** antimicrobial resistance, multi-drug-resistance, proteomics, mass spectrometry, ESKAPE pathogens

## Abstract

Antimicrobial resistance is an escalating global threat that undermines the efficacy of modern antibiotics and places a substantial economic burden on healthcare systems—costing Europe alone over EUR 11.7 billion each year due to rising medical expenses and productivity losses. While genomics and transcriptomics have significantly advanced our understanding of the genetic foundations of resistance, they often fail to capture the dynamic, real-time adaptations that enable bacterial survival. Proteomics, particularly mass spectrometry-based strategies, bridges this gap by uncovering the functional protein-level changes that drive resistance, persistence, and tolerance under antibiotic pressure. In this review, we examine how proteomic approaches provide new insights into resistance mechanisms across various antibiotic classes, with a particular focus on β-lactams, aminoglycosides, and fluoroquinolones, highlighting clinically relevant pathogens, especially members of the ESKAPE group. Finally, we examine future directions, including the integration of proteomics with other omic technologies and the growing role of artificial intelligence in resistance prediction, paving the way for more predictive, personalized, and effective solutions to combat antimicrobial resistance.

## 1. Introduction

Antimicrobial Resistance (AMR) occurs when microorganisms, including bacteria, fungi, viruses, and parasites, evolve to resist antimicrobial agents, including antibiotics, that were once effective [1,2,3]. This emerging global issue has become one of the greatest concerns of the 21st century, driven by the rapid rise in AMR infections and the insufficient development of new antimicrobial agents [4]. Often referred to as the “Silent Pandemic,” AMR is no longer a future threat but a present and urgent crisis requiring immediate action and more effective strategies [5]. The consequences of AMR are far-reaching, affecting not only developing countries but also global public health systems. Infections caused by resistant pathogens lead to more severe illness, extended hospitalizations, increased healthcare costs, higher expenses for second-line drugs, and higher rates of treatment failures [4,6]. In Europe alone, the economic impact of AMR is estimated to exceed EUR 11.7 billion annually, primarily due to increased healthcare expenditure and productivity losses in the workforce [7]. In 2019, AMR was associated with over 1.2 million deaths, and this number is expected to rise to approximately 10 million per year by 2050 if current trends persist [8,9].

Antimicrobials, particularly antibiotics, have been among the most impactful medical advancements in history, significantly reducing death rates from infectious diseases that once dominated the global health burden [10]. Since the introduction of antibiotics, life expectancy worldwide has increased by an average of 23 years [11]. However, despite the development of over 150 new antibiotics since the discovery of penicillin in 1928, which initiated the “golden era” of antibiotics, their overuse and misuse have accelerated the emergence of resistant and multi-drug-resistant strains, often referred to as “superbugs”, which significantly increase mortality rates [12]. In response to rising resistance, pharmaceutical companies have primarily developed “me-too” drugs—lightly altered versions of existing antibiotics that typically work through the same mechanism of action. These drugs are designed to be less toxic or more effective, but they only offer a limited delay in the development of bacterial resistance to a particular drug class [13].

**Figure 1:**
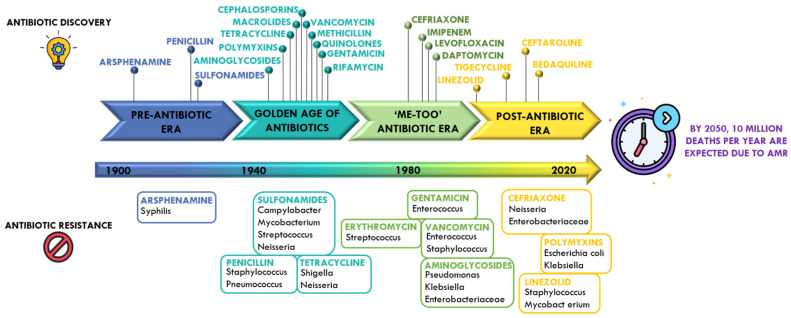
Timeline illustrating the discovery of key antibiotics and the subsequent development of resistance in different bacterial strains.

As illustrated in Figure 1, the antibiotic development pipeline is nearly exhausted, with the number of newly approved agents steadily declining over the past few decades [14]. A key driver of this stagnation is the systematic shift in pharmaceutical investment away from antibiotics and toward more profitable therapeutic areas, particularly chronic conditions such as diabetes and hypertension. This economic reallocation reflects the perceived unattractiveness of the antibiotic market, where returns on investment are significantly lower compared to long-term treatments or high-cost biologics [15,16,17]. Unlike drugs for chronic conditions, which offer sustained revenue over time, antibiotics are typically prescribed for short durations, and stewardship efforts aimed at limiting resistance further constrain their market potential. As a result, large pharmaceutical companies have progressively withdrawn from antibacterial R&D over the past two decades, leading to a shrinking pipeline of new antibiotics, particularly those with novel mechanisms of action. This has created a fragile innovation ecosystem, incapable of adequately responding to the rising global threat of AMR [17].

At the root of this crisis lies a systemic over-reliance on antibiotics, especially in sectors where regulation is lacking. Shockingly, 80% of antibiotics are administered to animals, often as growth promoters or prophylactic treatments in intensive farming operations, with only 20% reserved for human use [18].

**Figure 2:**
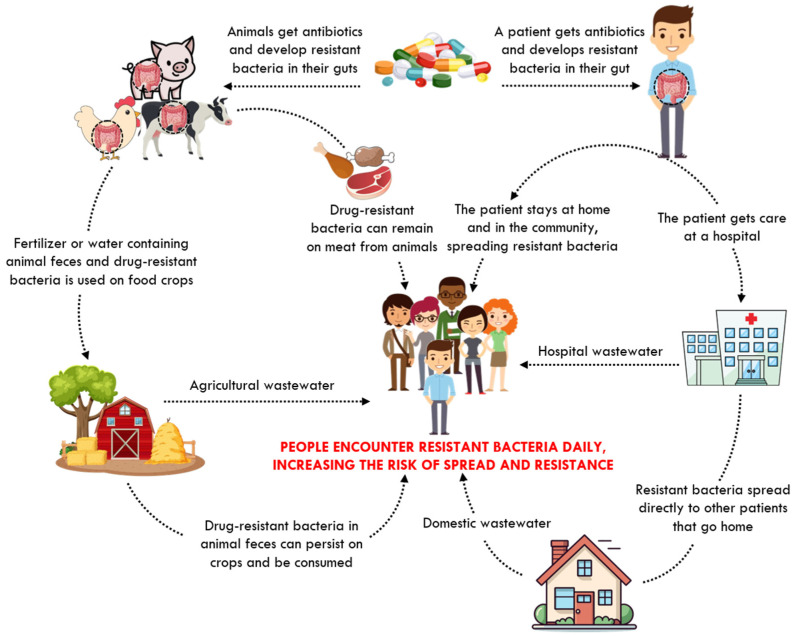
Spread of antibiotic resistance. Adapted from [23].

In addition to agricultural overuse, over-prescription of antibiotics in human healthcare exacerbates resistance. In some countries, antibiotics are sold over the counter without prescriptions; in other cases, antibiotics are frequently prescribed without laboratory confirmation of a bacterial infection, and sometimes even to treat viral illnesses, due to the absence of a definitive diagnosis—factors that contribute significantly to the acceleration of resistance [19,20,21,22]. The environmental release of antimicrobials and resistant organisms adds an additional layer of complexity. As shown in Figure 2, various sources contribute to environmental AMR contamination, creating a reservoir of resistant pathogens in agricultural and domestic settings.

Accurate identification and monitoring of these pathogens are essential to controlling the spread of resistance [24]. Accordingly, the development and application of precise, systematic analytical tools for the detection of antibiotic resistance mechanisms are a critical prerequisite for overcoming AMR.

Traditionally, antimicrobial susceptibility testing (AST) in clinical microbiology relies on phenotypic methods such as the minimum inhibitory concentration (MIC). More recently, whole-genome sequencing (WGS) has emerged as a powerful approach, providing comprehensive insights into the genetic potential of an organism. Several publicly available tools now facilitate AMR prediction from WGS data [25], with the Comprehensive Antibiotic Resistance Database (CARD) standing out for its high-quality, manually curated models based on experimentally validated genotype–phenotype associations [26]. CARD encompasses a range of resistance determinants, including protein homologs, rRNA variants, and regulatory mutations in efflux systems.

Nonetheless, WGS remains insufficiently validated for routine AST in clinical settings [27]. Although transcriptomics provides insight into gene expression at the mRNA level, it does not always accurately reflect the actual protein levels due to post-transcriptional regulation, variable protein stability, and translational control mechanisms [28,29]. Therefore, relying solely on mRNA measurements may lead to incomplete or misleading conclusions about cellular function and phenotype [30]. This limitation highlights the necessity of integrating proteomic data for a more comprehensive understanding of gene expression and protein function. Proteomics-based methods address this gap by quantifying resistance-associated proteins directly, offering the most definitive molecular evidence of AMR [31].

Proteomics enables the identification and quantification of the full complement of proteins (the proteome) in a given organism, tissue, or cell type. The proteome is inherently dynamic, influenced by intricate regulatory mechanisms that control protein expression [32], localization [33], activity, and structural modifications. These layers of control lead to substantial variability in the bacterial response to antibiotic pressure. As a result, proteomic technologies have become central to biomedical research on pathogens, offering insights that extend beyond the genetic blueprint. By examining protein-level responses, proteomics reveals how bacteria adapt to antibiotics and develop resistance, uncovering shifts in biological pathways related to resistance, virulence, and other adaptive traits [34].

In this review, we first distinguish among the distinct bacterial strategies of persistence, tolerance, and resistance. We then highlight how proteomics serves as a powerful tool to investigate these phenotypes and examine the molecular mechanisms underlying resistance to widely used antibiotics, specifically β-lactams, aminoglycosides, and fluoroquinolones. Finally, we outline our perspective on future research directions in this evolving field.

## 2. Different Survival Strategies of Bacteria Against Antibiotics

**Figure 3:**
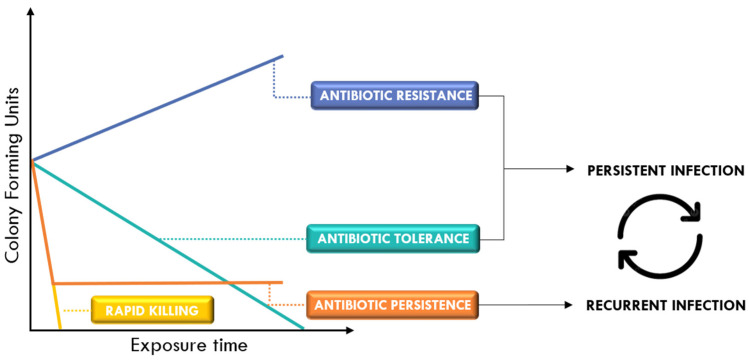
Schematic illustration highlighting the differences between antibiotic resistance, tolerance, and persistence.

Bacteria use different strategies to withstand antibiotic treatment, primarily through three distinct molecular mechanisms: resistance, persistence, and tolerance (Figure 3). Resistant cells continue to grow in the presence of antibiotics, which sets them apart from tolerant or persistent cells. Tolerance refers to a reduced susceptibility across the bacterial population, resulting in slower bacterial killing and requiring prolonged antibiotic exposure to achieve eradication. Persistence, on the other hand, is characterized by the survival of a small subpopulation of highly tolerant cells. This phenomenon results in a biphasic killing curve, where an initial rapid decline reflects the elimination of the majority of susceptible cells, followed by a slower killing phase during which these persister cells remain viable and tolerant to antibiotic treatment. Among these survival strategies, resistance is the most well-characterized and will be the primary focus of this review.

Resistance typically arises from genetic mutations or the acquisition of resistance genes, which enable bacteria to neutralize the effects of antibiotics and survive and proliferate despite high drug concentrations [35]. Unlike tolerance and persistence, resistance is transmissible: it can spread not only through vertical transmission during replication but also via horizontal gene transfer of mobile genetic elements, facilitating rapid dissemination within and across species [36].

**Figure 4:**
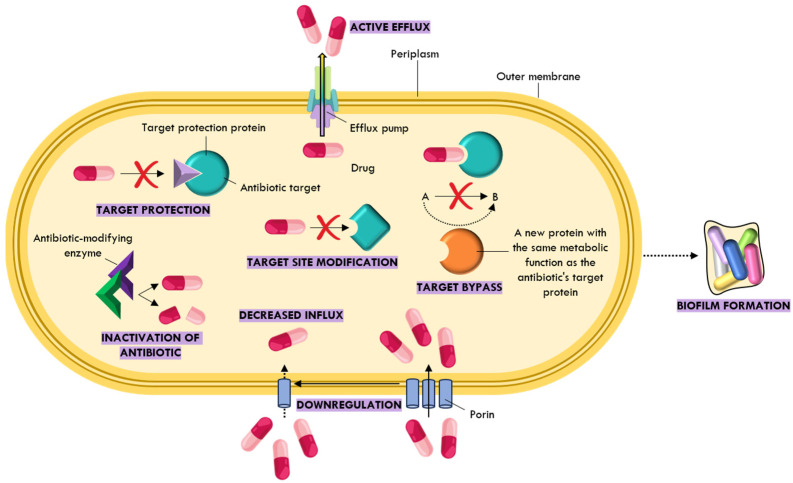
Overview of the molecular mechanisms of antibiotic resistance. The red “X” symbols highlight specific points where bacterial resistance directly disrupts the interaction between the antibiotic and its target, emphasizing the mechanisms that block antibiotic action at the target level.

Over time, bacteria have evolved a wide array of molecular mechanisms to counteract antibiotic activity. These include enzymatic degradation or modification of antibiotics, reduced uptake through changes in membrane permeability, metabolic rewiring, alteration or protection of antibiotic targets, and increased drug efflux [37,38]. Enzymatic degradation involves the hydrolysis of specific functional groups, rendering antibiotics inactive [39]. In contrast, modifying enzymes attach chemical groups—such as acetyl, phosphate, or adenyl groups—to the antibiotic molecule, thereby preventing its interaction with the target site [40]. Target site alterations typically result from mutations or enzymatic modifications that reduce the antibiotic’s binding affinity to its cellular target, such as the bacterial ribosome [41,42]. In target bypass, bacteria produce alternative proteins that can carry out the same function as the original target, effectively negating the antibiotic’s action [43]. In addition, bacteria may limit antibiotic entry by altering or downregulating porins—membrane proteins that facilitate the passive transport of molecules into the cell [44], thereby reducing outer membrane permeability [44]. They may also enhance active efflux mechanisms, in which transmembrane pumps expel antibiotics to reduce their intracellular concentration [45]. Another resistance strategy, known as target protection, involves proteins that bind to and shield the antibiotic target, thereby preventing inhibition [46]. These various resistance mechanisms are summarized in Figure 4.

In addition to understanding the molecular mechanisms that enable bacterial resistance, it is crucial to consider the biological trade-offs associated with these adaptations. Fitness costs linked to AMR represent a critical trade-off with significant implications for human health. It is widely believed that, in the absence of antibiotics, AMR pathogens experience reduced fitness, which may manifest as diminished competitive ability [47,48], slower growth rates [49,50,51], and/or decreased virulence [50] This assumption forms the basis of public health strategies aimed at limiting antibiotic use to curb resistance prevalence [52], relying on the expectation that costly resistance mutations will decrease in frequency once selective pressure is lifted. Supporting this view, experimental studies investigating the fitness impacts of individual AMR determinants, whether chromosomal mutations or mobile genetic elements, generally identify associated fitness costs, although some resistance mutations appear to incur little or no fitness penalty [48,53]. Understanding these fitness trade-offs is critical, as they shape the evolutionary trajectories of resistance and influence the persistence and spread of AMR pathogens in clinical and environmental settings.

In addition to the mentioned molecular mechanisms, bacteria employ structural strategies. Bacteria can also form biofilms—structured surface-associated communities with heterogeneous nutrient access and reduced antibiotic penetration—which further protect them from antimicrobial agents [54].

Over the past few decades, various microorganisms have developed AMR through different mechanisms [55]. Methicillin-resistant *Staphylococcus aureus* (MRSA), for example, acquires resistance to β-lactam antibiotics through the horizontal transfer of the mecA or mecC genes, located on the staphylococcal cassette chromosome mec (SCCmec). Additional mutations, such as those affecting PBP2a, have also been associated with resistance to advanced β-lactams like ceftaroline [56]. Carbapenem-resistant *Enterobacteriaceae*, such as *Klebsiella pneumoniae* and *Escherichia coli*, have gained carbapenemase-encoding plasmids that facilitate resistance and rapid spread [57]. Extended-spectrum β-lactamase (ESBL)-producing *E. coli* are similarly resistant to penicillins and cephalosporins via plasmid-mediated gene acquisition. Multidrug-resistant *Mycobacterium tuberculosis* strains harbor chromosomal mutations that confer resistance to first-line anti-tuberculosis drugs [58]. *Acinetobacter baumannii* combines acquired genes and mutations to develop broad-spectrum resistance, while *Neisseria gonorrhoeae* has become increasingly resistant and challenging to treat due to resistance to multiple classes of antibiotics [59]. Fungal pathogens such as *Candida* species have also developed resistance, particularly to fluconazole, complicating treatment in immunocompromised populations [60,61].

## 3. Proteomics by Mass Spectrometry as a Tool for Understanding AMR

With the increasing prevalence of antibiotic resistance genes in pathogenic bacteria, proteomic analysis has become essential for evaluating dynamic, system-wide changes in protein expression. Unlike genomic or transcriptomic approaches, proteomics offers biologically relevant insights by reflecting the actual biological state of the cell, including post-translational modifications and protein turnover [62].

Mass spectrometry (MS) has become a cornerstone in modern proteomics due to its exceptional sensitivity, specificity, and ability to characterize proteins at low concentrations [63,64]. MS works by ionizing samples and separating the ions based on their mass-to-charge (*m*/*z*) ratio, generating a mass spectrum. Since the direct injection of complex biological samples into a mass spectrometer is often inefficient and can hinder accurate analysis, a prior separation step is usually required. This involves isolating proteins or peptides using separation techniques like two-dimensional gel electrophoresis (2DE), SDS-PAGE, and liquid chromatography (LC), which help reduce sample complexity and improve detection sensitivity during MS analysis.

Ionization methods such as electrospray ionization (ESI) and matrix-assisted laser desorption/ionization (MALDI) are commonly used. Mass analyzers, including ion trap, quadrupole, time-of-flight (TOF), and Fourier transform ion cyclotron resonance (FT-ICR), provide both structural and quantitative data. TOF instruments, with high acquisition rates, are particularly useful when combined with ion mobility spectrometry (IMS), which separates ions by size, shape, or collisional cross-section (CCS) [65,66,67]. When coupled with LC and MS, IMS–MS introduces a fourth analytical dimension, enhancing resolution and speed [66,68,69,70].

Technological advances, especially tandem MS (MS/MS), have significantly increased the depth and accuracy of proteomic analyses. De novo peptide sequencing, enabled by fragmentation techniques such as collision-induced dissociation (CID) and higher-energy collisional dissociation (HCD), allows sequence identification even without a reference protein database [71,72,73]. Despite challenges such as incomplete ion series coverage, methods like peptide sequence tags [74] and database search algorithms (e.g., SEQUEST [75], MASCOT, Crux [76], Comet [77], Andromeda [78]) have made high-throughput protein identification widely accessible.

MS-based quantification can be achieved through label-based or label-free approaches. Label-based methods involve incorporating stable isotopes during sample preparation, such as ^18^O labeling [79], chemical dimethyl tagging [80], metabolic labeling (SILAC) [81,82], and isobaric tags like iTRAQ [34]. These techniques allow precise quantification but may be limited by cost and scalability.

In contrast, label-free quantification (LFQ) uses MS signal intensities or spectral counting to assess peptide abundance post-acquisition, without the need for isotopic labeling [83,84]. LFQ is more suitable for large-cohort studies and cost-sensitive or resource-limited settings, although it demands high reproducibility in sample handling. Advanced software tools support LFQ normalization and analysis [85,86].

**Figure 5:**
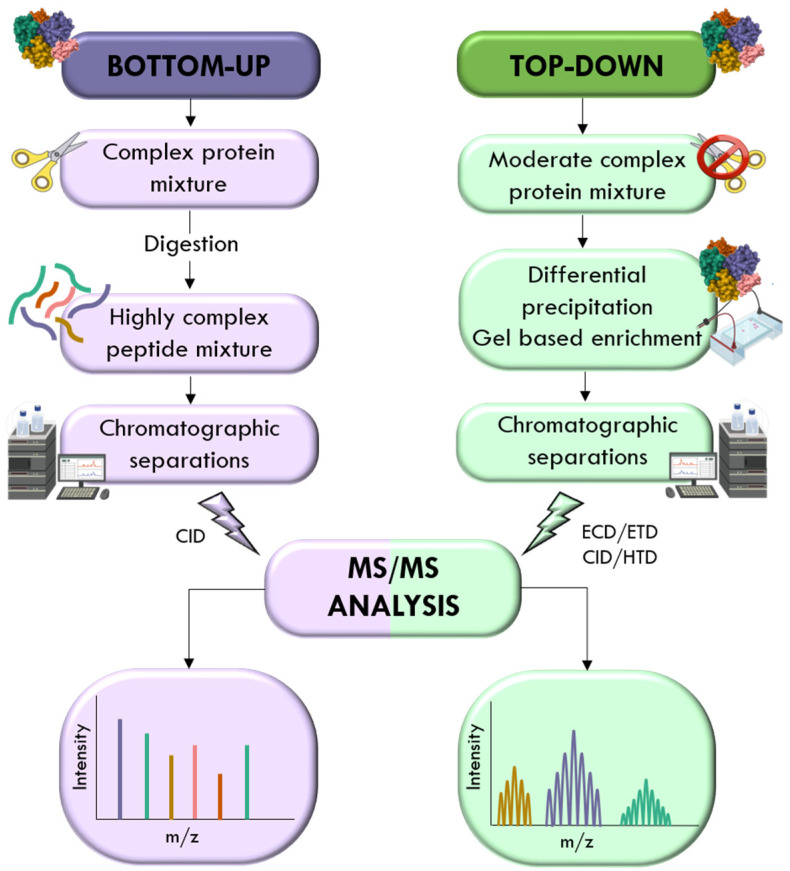
Comparative overview of top-down and bottom-up proteomics workflows used in MS-based protein analysis.

Proteomic analysis is typically categorized into two primary approaches: “top-down” and “bottom-up,” as illustrated in Figure 5.

Top-down proteomics analyzes intact proteins, making it particularly effective for characterizing co-existing and site-specific post-translational modifications (PTMs) within a single proteoform [87,88]. This approach retains high-level structural information and eliminates ambiguity associated with protein inference from peptides [89]. However, the method demands high resolution in the mass-to-charge (*m*/*z*) ratio and may require charge state stabilization. To enhance accuracy and resolve proteoform complexity, top-down workflows often incorporate tandem MS techniques to fragment and sequence intact proteins, enabling precise localization of PTMs and isoforms [87].

In contrast, bottom-up proteomics digests proteins into peptides prior to analysis, facilitating simpler and more consistent chromatographic separation. Due to reduced variability in chemical properties such as hydrophobicity and isoelectric point, shorter peptides are more amenable to reversed-phase ultra-high-performance liquid chromatography (RP-UHPLC), which typically employs a C18 stationary phase and a water-acetonitrile gradient. This method is broadly applicable to diverse biological matrices, including urine [90], blood [91], cerebrospinal fluid [92], bacterial biofilms [93], cell lysates [94], soil [95], and wastewater [96].

The general bottom-up workflow includes sample homogenization, protein extraction, enzymatic digestion (usually with trypsin), desalting, and MS analysis. Peptides are typically subjected to a full MS scan followed by multiple MS/MS (tandem) scans for structural characterization and quantification.

**Figure 6:**
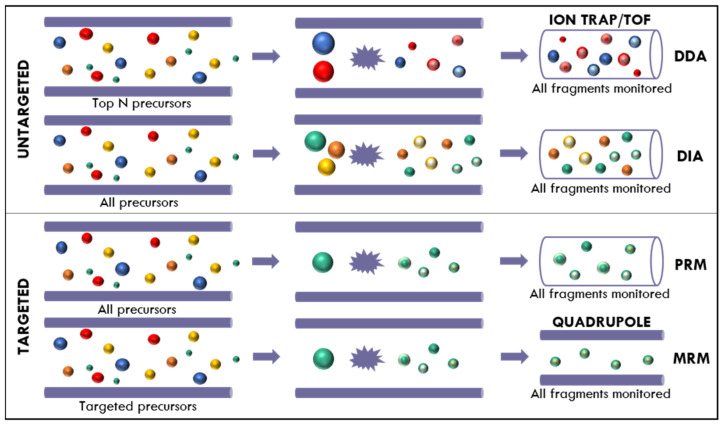
A schematic showing the process of peptide isolation, fragmentation, and analysis by a mass spectrometer operating in data-dependent acquisition (DDA), multiple reaction monitoring (MRM), parallel reaction monitoring (PRM), or data-independent acquisition (DIA). Adapted from [101].

As illustrated in Figure 6, MS-based proteomics can be conducted using either untargeted (discovery-based) or targeted approaches. Shotgun proteomics, an untargeted strategy, utilizes Data-Dependent Acquisition (DDA) to detect and quantify thousands of peptides in a single run. DDA dynamically selects the most intense precursor ions for fragmentation, which can introduce variability due to stochastic sampling. Despite generating rich peptide spectral libraries, DDA may underrepresent low-abundance peptides.

To address this limitation, Data-Independent Acquisition (DIA) systematically fragments all ions across defined *m*/*z* windows, providing greater reproducibility and more comprehensive proteome coverage. However, DIA often relies on pre-established DDA libraries, posing challenges for novel proteoforms or personalized antigens [97].

Targeted proteomics offers enhanced sensitivity and reproducibility for quantifying specific peptides. Selected Reaction Monitoring (SRM) and Multiple Reaction Monitoring (MRM), typically carried out on triple quadrupole instruments, are widely employed for high-throughput validation of known targets. These methods can detect multiple isoforms and PTMs with exceptional specificity in a single run [98,99,100]. A more recent approach, Parallel Reaction Monitoring (PRM), follows a similar targeted acquisition strategy but uses high-resolution detection to improve selectivity and confidence in peptide identification.

New developments in Data-Independent Acquisition (DIA), particularly Sequential Window Acquisition of All Theoretical Mass Spectra (SWATH-MS), have revolutionized proteomics by merging the strengths of both targeted and untargeted approaches. SWATH-MS enables high-throughput, quantitative, and reproducible analysis within a single experimental run by fragmenting all precursor ions across sequential *m*/*z* windows [98,102,103,104]. Its capacity for comprehensive proteome coverage and accurate quantification has made it particularly valuable across various biomedical and microbiological applications. These include drug and vaccine development [105], biomarker discovery in HIV-1 [106], characterization of tissue-specific protein changes during sepsis [107], and in-depth analysis of proteomic adaptations under antibiotic pressure [108]. Notably, SWATH-MS has also provided novel insights into host–pathogen interactions and neurological responses in bacterial meningitis [109].

As previously mentioned, the integration of ion mobility spectrometry (IMS) into MS workflows adds an additional separation dimension based on collision cross-section (CCS), offering valuable structural insights about peptide and protein conformation [88,110]. This fourth analytical dimension enhances both the resolution and confidence of proteomic identifications, particularly in complex biological samples.

In structural proteomics, crosslinking mass spectrometry (XL-MS) is an important technique that uses bifunctional crosslinking agents to covalently bind spatially proximal amino acid side chains within or between proteins. This covalent labeling approach preserves native protein structures and interactions. After digestion and MS analysis, the crosslinked peptide pairs provide distance constraints that can be translated into three-dimensional models of protein complexes [111]. XL-MS is particularly useful in mapping conformational changes, studying protein–protein interactions, and modeling large macromolecular assemblies under different physiological or stress conditions, including antibiotic treatment.

Beyond their mechanistic value, proteomic signatures have shown promise as diagnostic and prognostic biomarkers in AMR surveillance. For instance, the consistent upregulation of efflux-related proteins or specific β-lactamases could support targeted diagnostic development. Furthermore, proteomic profiles offer a rational basis for combinatorial therapy, by identifying stress pathways or metabolic bottlenecks that may be exploited pharmacologically to restore antibiotic susceptibility. Table 1 provides a summary of recent key studies that illustrate the diverse applications of proteomics in AMR research—from mechanistic investigations to therapeutic development—which, while relevant, were not discussed in detail in the main body of the review.

### 3.1. Antimicrobial Resistance in ESKAPE Pathogens

Many resistant infections in clinical settings are due to ‘ESKAPE’ pathogens: *Enterococcus faecium*, *Staphylococcus aureus*, *Klebsiella pneumoniae*, *Acinetobacter baumannii*, *Pseudomonas aeruginosa*, and other members of the *Enterobacterales* family [129]. These microorganisms are particularly adept at thriving in healthcare environments. They are characterized by a wide range of intrinsic and acquired resistance mechanisms, making them major contributors to the emergence, persistence, and global spread of AMR. Due to their clinical relevance and adaptability, ESKAPE pathogens have become key models for studying antibiotic resistance in both hospital and community settings. These bacteria acquire resistance through spontaneous mutations and, more importantly, via the uptake of mobile genetic elements (MGEs), which facilitate the horizontal transfer of resistance genes across species and genera [130]. ESKAPE organisms are often resistant to multiple antibiotic classes, including oxazolidinones, lipopeptides, macrolides, fluoroquinolones, tetracyclines, β-lactams, and β-lactam/β-lactamase inhibitor combinations. Alarmingly, they also exhibit reduced susceptibility to last-resort treatments such as carbapenems, glycopeptides, and polymyxins [131]. Despite the development of various treatment strategies and ongoing clinical trials investigating vaccine candidates, no vaccines are currently available to prevent infections caused by ESKAPE pathogens [132]. Their MDR profiles significantly increase the risk of severe disease outcomes and mortality. In many cases, AMR-related infections also lead to additional complications, including complications resulting from medical interventions [133].

Although taxonomically diverse, ESKAPE organisms share a number of resistance-associated traits, most notably their capacity to form biofilms on biotic and abiotic surfaces. This biofilm formation enhances their survival and persistence in clinical settings, contributing to chronic infections and reduced antibiotic efficacy. Combating AMR in these pathogens requires a multifaceted approach. Alongside the discovery of new therapeutic agents and the prudent use of existing antibiotics, there is an urgent need to develop advanced and rapid diagnostic tools.

Moreover, AMR in ESKAPE pathogens is not limited to clinical environments. These organisms are also commonly found in the environment, particularly in livestock and in wastewater from agricultural and food-processing facilities. Veterinary outbreaks have led to the detection of resistant strains such as MRSA, vancomycin-resistant *Enterococcus* (VRE), and ESBL-producing *Escherichia coli* and *Klebsiella pneumoniae* [134].

These findings underscore the One Health dimension of AMR, linking human, animal, and environmental health.

In the following sections, we will examine the molecular mechanisms of resistance to three major classes of antibiotics—β-lactams, aminoglycosides, and fluoroquinolones—with particular emphasis on findings from proteomic studies. Special attention will be given to *E. coli* and ESKAPE pathogens, which remain at the forefront of AMR research due to their clinical impact and proteomic complexity.

#### 3.1.1. Resistance to β-Lactams

β-Lactam antibiotics are a broad class of antibiotics and are currently among the most widely used drugs for treating bacterial infections [135]. These antibiotics are primarily categorized based on their chemical structure into penicillins, cephalosporins, carbapenems, monobactams, and a few other minor categories. β-Lactams work by disrupting the synthesis of the bacterial cell envelope, which destabilizes the cell’s integrity, leading to a loss of selective permeability and, ultimately, cell death [136].

Gram-positive bacteria resist β-lactams through multiple mechanisms, including the production of β-lactamases that break down the β-lactam ring, acquisition of alternative penicillin-binding proteins (PBPs) enabling target bypass, target site alterations, and the use of L,D-transpeptidases [137,138]. Gram-negative bacteria, on the other hand, typically resist β-lactams by producing β-lactamases, rendering the antibiotic ineffective [137,138,139].

In addition to this, they often exhibit changes in porin expression. Porins are β-barrel proteins in the outer membrane of Gram-negative bacteria that allow small polar molecules, including many antibiotics, to enter the cell. Altered or reduced porin expression can limit the antibiotic’s access to the bacterial cell [140]. Moreover, some resistant strains increase the activity of efflux pumps, which actively expel toxic substances, including antibiotics, from the periplasmic space, further contributing to resistance [44].

Early proteomic studies by Xu et al. and Peng et al. used 2DE to investigate β-lactam resistance mechanisms in *E. coli* and *P. aeruginosa*, focusing on responses to ampicillin (AMP) and non-β-lactam antibiotics like tetracycline (TC) and kanamycin (KN) [141,142]. In *E. coli*, eight proteins were differentially expressed under AMP treatment—seven were upregulated, including porins OmpC, OmpW, Tsx, and the efflux pump component TolC, while BamC was downregulated. Although β-lactam resistance is typically associated with porin downregulation to reduce drug uptake [143,144], the authors proposed that increased porin expression might aid antibiotic transport into outer membrane vesicles, where β-lactamase can hydrolyze the drug before it reaches the cell wall [145]. Notably, *E. coli* showed a similar proteomic response to both AMP and TC. *P. aeruginosa*, in contrast, exhibited antibiotic-specific regulation of porins (e.g., OprD, OprG), highlighting a species-specific adaptation involving unique outer membrane restructuring.

Building on this, Ude et al. demonstrated that alternative uptake routes, such as passive diffusion or transport via outer membrane proteins like BamA, can complement or replace porin-mediated transport for certain antibiotics [146]. Yet, porins remain essential for the uptake of carboxylated compounds, delineating a nuanced, compound-specific permeability model.

In *Acinetobacter baumannii*, a notorious MDR nosocomial pathogen, proteomic and glycoproteomic analyses by Wang et al. identified extensive upregulation of proteins involved in membrane stabilization, stress response, and efflux in MDR strains compared to drug-susceptible counterparts [147]. Notably, glycosylation of ATP emerged as a unique signature of MDR isolates, suggesting potential post-translational regulation of metabolic or transport pathways. Complementing this, Hillyer et al. uncovered β-lactam-specific expression of diverse β-lactamase isoforms—predominantly Class C—underscoring the adaptive plasticity of the *A. baumannii* resistome in response to subtle chemical variations among β-lactams [148].

Proteomic insights into MRSA, as presented by Liu et al., further exemplify the complexity of β-lactam resistance in Gram-positive bacteria. Their study found that β-lactamase was upregulated in MRSA and, as expected, the resistance hallmark PBP2a was also induced in MRSA strains only. In contrast, both MRSA and methicillin-susceptible *S. aureus* (MSSA) activated metabolic pathways related to peptidoglycan biosynthesis and CoA metabolism under oxacillin stress [149]. Importantly, co-treatment with the erythromycin derivative SIPI-8294 amplified bacterial killing, correlating with redox imbalance and cell wall disruption, as revealed by proteomics [150,151].

Finally, in carbapenem-resistant *Klebsiella pneumoniae* (NDM-4), Sharma et al. identified over 50 proteins that were upregulated during meropenem exposure, including those related to β-lactam cleavage, the translation machinery, and DNA/RNA modification [152]. These findings highlight the complex and multifaceted protein-level responses contributing to carbapenem resistance in *K. pneumoniae*. Together, these studies illustrate that β-lactam resistance is not a monolithic phenomenon, but rather a dynamic, species- and antibiotic-specific process. While common themes, such as β-lactamase production, porin modulation, and efflux pump activation, emerge across bacterial taxa, each pathogen deploys a unique proteomic toolkit shaped by its ecological niche and structural biology. Advances in proteomics have revealed the complex mechanisms by which bacteria evade β-lactam antibiotics, revealing interconnected resistance pathways and pointing to strategies such as combination therapies and metabolic disruption.

#### 3.1.2. Resistance to Aminoglycosides

Aminoglycosides, such as gentamicin, kanamycin, amikacin, and streptomycin, are broad-spectrum antibiotics that disrupt bacterial protein synthesis by binding the 16S rRNA of the 30S ribosomal subunit, causing mistranslation and ultimately cell death [153]. Their uptake depends on electrostatic interactions with the bacterial membrane and requires energy from the proton-motive force [154]. Resistance mechanisms are varied and include enzymatic modifications (such as acetylation, phosphorylation, and adenylylation) [155], changes in membrane permeability [156,157], reduced proton-motive force, enhanced efflux, and ribosomal mutations [158].

Proteomic studies have deepened our understanding of these resistance strategies, uncovering novel regulatory, structural, and metabolic adaptations [159].

In *Escherichia coli*, proteomic analysis by Zhang et al. revealed that MipA, a cell envelope-associated protein, was significantly downregulated in kanamycin-resistant strains [160]. Its deletion further increased resistance, suggesting that MipA supports antibiotic susceptibility, potentially through its role in maintaining outer membrane permeability. Moreover, while MipA itself is not directly involved in protein folding, it may act in coordination with systems such as the BAM complex, which is essential for the folding and insertion of β-barrel outer membrane proteins [161].

*Pseudomonas aeruginosa* adapts to aminoglycoside stress via induction of proteostasis networks. Wu et al. used time-resolved proteomics to demonstrate that exposure to tobramycin triggered a strong upregulation of heat shock proteins, particularly IbpA, at high antibiotic concentrations. Aminoglycosides induce cell death by binding to the 30S ribosomal subunit, leading to mistranslation and accumulation of misfolded proteins, which cause proteotoxic stress and membrane damage. In this context, IbpA and other heat shock proteins act as molecular chaperones that prevent aggregation of aberrant proteins and help maintain protein homeostasis. Although single deletion of IbpA had little impact, combined deletions with other heat shock proteins increased susceptibility to aminoglycosides, suggesting functional redundancy among these stress response elements [162]. Additionally, membrane proteomics of a kanamycin-resistant strain highlighted enhanced efflux and reduced permeability as central resistance strategies [142].

In *Edwardsiella tarda*, Ye et al. discovered that exogenous alanine sensitizes bacteria to kanamycin by multiple mechanisms [163]. Proteomic analysis identified upregulation of OmpA, NagE, and FadL—proteins involved in nutrient transport and membrane-associated functions. Deletion of NagE and FadL led to lower intracellular kanamycin levels, demonstrating their involvement in antibiotic influx. Simultaneously, alanine treatment increased reactive oxygen species (ROS) production while suppressing antioxidant systems, creating a pro-oxidative environment that enhanced aminoglycoside efficacy.

*Acinetobacter baumannii* adds another layer to resistance via phosphorylation-based regulation. Soares et al. performed phosphoproteomic profiling and found 201 phosphorylation sites across a range of resistance- and stress-related proteins [164]. These included a streptomycin 3-adenylyltransferase, whose phosphorylation may regulate enzymatic inactivation of antibiotics, and other phosphoproteins such as RecA, an ABC transporter, and superoxide dismutase, indicating tight post-translational control of stress and resistance pathways [163]. By integrating proteomics and functional validation, researchers have unveiled both conserved and species-specific strategies that bacteria use to counteract aminoglycosides.

#### 3.1.3. Resistance to Quinolones

Quinolones are fully synthetic, broad-spectrum bactericidal agents with activity against a wide range of Gram-positive and Gram-negative bacteria [165]. Fluoroquinolones, the most widely used subclass of quinolones, exert their antimicrobial effect by targeting bacterial DNA replication enzymes—primarily DNA gyrase and topoisomerase IV. These enzymes are essential for DNA supercoiling and decatenation, respectively. Fluoroquinolones often inhibit both targets within the same organism. However, the relative contribution of each enzyme to drug lethality may vary depending on the species, growth conditions, and specific compound used [166,167,168]. In addition to their direct inhibition of DNA gyrase and topoisomerase IV, fluoroquinolones have also been shown to stimulate the generation of ROS as a downstream consequence of DNA damage, further contributing to their bactericidal activity [169,170].

Resistance to quinolones typically arises from point mutations in the quinolone resistance-determining regions (QRDRs) of the gyrA and parC genes, which reduce drug binding affinity to DNA gyrase and topoisomerase IV, respectively [171]. However, bacteria also employ a variety of non-mutational strategies to evade fluoroquinolone action, including reduced outer membrane permeability, overexpression of multidrug efflux pumps, and the acquisition of plasmid-mediated qnr genes that protect target enzymes and widen the mutant selection window.

Recent proteomic studies have revealed that quinolone resistance is not simply a matter of enzyme evasion but involves broader cellular remodeling, metabolic suppression, and regulatory network shifts.

In *E. coli*, the downregulation of outer membrane protein LamB has been associated with reduced ciprofloxacin permeability [172]. LamB deletion mutants, as shown by Li et al., also exhibit impaired central carbon metabolism—including the TCA cycle, glycolysis, and pentose phosphate pathway—and downregulated translation machinery, suggesting that resistance may involve a metabolic slowdown strategy that conserves energy and reduces cellular targets for the drug [173].

Beyond permeability and metabolism, enzyme-level adaptation has also been observed. L-asparaginase was upregulated in extended-spectrum β-lactamase (ESBL)-producing, ciprofloxacin-resistant *E. coli* upon drug exposure, hinting at stress-linked nitrogen metabolism pathways as an unexplored resistance factor [174]. Similarly, in *Pseudomonas aeruginosa*, multiple proteomic studies underscore a phased resistance evolution. Su et al. demonstrated that adaptive evolution under ciprofloxacin stress increases phosphorylation of energy-related enzymes such as MMSADH and SSADH, which are likely involved in ROS detoxification and redox homeostasis [175].

Further, comparative proteomics by Peng et al. revealed that low-level resistant strains primarily activate oxidative stress responses and general metabolic shifts, whereas high-level resistant mutants show overexpression of the MexCD-OprJ efflux system and suppression of PQS quorum-sensing, suggesting a shift from adaptive resilience to dedicated resistance pathways [176].

The biofilm phenotype also contributes to quinolone resistance. Prolonged ciprofloxacin exposure, as shown by Machado et al., triggers the expression of phage-associated proteins in *P. aeruginosa*, enhancing biofilm heterogeneity and persistence—a key strategy for surviving antimicrobial stress [177].

Other organisms exhibit distinct but overlapping responses. In *Vibrio alginolyticus*, levofloxacin resistance involved suppressed energy metabolism and membrane potential, reflecting a resource-conservation state [178]. In *Salmonella Typhimurium*, Correia et al. identified resistance-associated rewiring of Fur-regulated iron homeostasis, two-component regulatory systems, and changes in membrane proteins (e.g., OmpC, OmpD, OmpX, MipA) and aminoglycoside-modifying enzyme AAC(6′)-Ib-cr4, pointing to a multi-systemic stress adaptation involving both permeability and intracellular detoxification [160,179].

Advanced proteomic techniques such as COFRADIC (combined fractional diagonal chromatography) have enabled finer resolution of resistance at the proteoform level. Using this method, Vranakis et al. found that *Coxiella burnetii* resistant strains upregulated glutathione S-transferase (GST) and FabZ, proteins associated with detoxification and membrane remodeling, reinforcing conserved themes of redox and envelope modification in resistance [180,181,182].

Finally, post-translational modifications such as lysine acetylation have emerged as regulators of quinolone resistance. In ciprofloxacin-resistant *Salmonella*, Li et al. identified differential acetylation of proteins related to antimicrobial defense, suggesting that acetylation fine-tunes protein activity in response to drug-induced stress and plays a role in phenotypic resistance evolution [183].

The growing body of proteomic evidence underscores that quinolone resistance is a systems-level phenomenon, driven by more than point mutations in DNA replication enzymes. Understanding these mechanisms in a species- and context-specific manner not only clarifies the biology of resistance but also illuminates novel targets for therapeutic intervention—whether by disrupting stress responses, modulating metabolism, or targeting resistance-associated proteoforms.

## 4. Advancing AMR Prediction: Future Directions and Challenges

Proteomics has established itself as a fundamental tool for unraveling the complex molecular mechanisms that drive AMR, offering a functional perspective that effectively bridges the gap between genetic information and phenotypic expression. Unlike genomics and transcriptomics, which provide static or indirect insights, proteomics directly captures the biological effectors of resistance—namely proteins—whose abundance, localization, and post-translational modifications are dynamically modulated in response to antibiotic exposure [31,32,62]. To fully understand the intricacies of AMR, it will be essential to integrate proteomics with other omic disciplines, including genomics, transcriptomics, and metabolomics. This multi-layered approach enables a more holistic, systems-level understanding of resistance, allowing researchers to uncover complex regulatory networks and metabolic interdependencies that remain obscured when each omic layer is studied in isolation [184]. Several recent studies have demonstrated the power of multi-omics integration in elucidating AMR mechanisms. For instance, Zampieri et al. combined transcriptomic, proteomic, and metabolomic data to map drug-induced metabolic rewiring in *E. coli*, revealing adaptive cross-talk between antibiotic stress responses and core metabolism [185]. Similarly, Zhou et al. employed an integrative approach combining genomics, transcriptomics, and metabolomics to investigate small colony variants of *Staphylococcus aureus* induced by sulfamethoxazole-trimethoprim treatment. Their analysis uncovered metabolic adaptations and transcriptional responses associated with resistance and virulence, providing new insights into AMR development [186]. Such integration holds significant promise not only for elucidating resistance mechanisms but also for identifying novel therapeutic targets, tracking the evolution of resistance, and discovering clinically relevant biomarkers that can inform diagnostics and personalized treatment strategies.

In parallel, recent advancements in artificial intelligence (AI) and machine learning (ML), particularly the application of deep learning (DL) frameworks such as MSDeepAMR and models based on MALDI-TOF spectral profiles, have opened new frontiers in AMR prediction. These tools are proving increasingly effective in classifying resistance phenotypes with high accuracy, thereby enabling faster diagnostics and more efficient surveillance systems [187,188,189,190,191]. While these developments underscore the transformative potential of AI-driven approaches [192,193], several challenges persist. The robustness and interpretability of ML outputs depend heavily on the availability and standardization of high-quality datasets, and clinical implementation is further constrained by regulatory, infrastructural, and interoperability barriers [194,195,196].

Continued progress in the field will also hinge on refining and standardizing proteomic technologies to ensure reproducibility across different laboratories and analytical platforms. The development of high-throughput, clinically adaptable workflows, as well as the expansion of curated, AMR-specific proteomic databases, will enhance both research applications and clinical utility. Moreover, longitudinal studies in clinical cohorts are needed to monitor resistance dynamics over time and under therapeutic pressure, enabling the identification of temporally regulated biomarkers and adaptive responses [197,198,199].

Equally important is the advancement of proteoform-resolved analyses, which allow the detection of specific isoforms or post-translationally modified variants that may drive resistance phenotypes—molecular features that often go undetected at the genomic level.

## 5. Conclusions

AMR constitutes a critical global health challenge with far-reaching clinical, economic, and societal implications. Proteomics has emerged as a powerful and functionally informative platform that complements genomic and transcriptomic data by capturing the dynamic and post-translational nature of bacterial responses to antibiotic pressure. MS-based proteomics offers unprecedented resolution in profiling resistance-associated proteins, identifying diagnostic biomarkers, and uncovering novel therapeutic targets. Its application to clinically relevant pathogens has deepened our understanding of resistance mechanisms and revealed complex regulatory adaptations that go beyond genetic mutations alone. Despite these advances, several challenges remain. Standardization of proteomic workflows, improved integration with other omic layers, and the development of robust, high-quality reference databases are essential for translating research findings into clinical practice. Furthermore, the incorporation of AI-driven tools holds great promise for enhancing the accuracy and speed of AMR diagnostics but will require careful validation, regulatory oversight, and infrastructure development. Looking ahead, the future of AMR research lies in comprehensive, multi-omic investigations across diverse clinical and environmental contexts. By bridging molecular insights with clinical outcomes, proteomics can contribute not only to a deeper mechanistic understanding of resistance but also to the development of precision medicine strategies and more effective public health interventions.

## Figures and Tables

**Table 1:** Summary of recent key studies illustrating the diverse applications of proteomics in AMR research.

Article	Year	Bacterial Strain(s)	Antibiotic(s) Involved	Key Proteins Identified	Affected Metabolic Pathways
[112]	2025	*Escherichia coli*	Ciprofloxacin, Enrofloxacin	Proteins linked to SOS response and RecA-independent mechanisms	SOS response, DNA repair, oxidative stress response, nucleotide metabolism
[113]	2025	*Escherichia* coli drug sensitive and MDR	Various antibiotics	A total of 763 differentially expressed proteins	Protein biosynthesis, transcription, translation, stress adaptation
[114]	2024	*Staphylococcus aureus* MRSA and MSSA	Methicillin	A total of 407 differentially expressed proteins	Fatty acid degradation, glycine, serine, and threonine metabolism
[115]	2023	*Stutzerimonas stutzeri*	Chloramphenicol, Minocycline	Multidrug/solvent RND membrane fusion protein, MexE	Membrane transport, antibiotic efflux
[116]	2023	*Pseudomonas aeruginosa*	Aztreonam, Carbenicillin, Piperacillin, Tobramycin	Various stress response proteins	Oxidative stress response, protein synthesis, biofilm formation
[117]	2023	*Acinetobacter baumannii*	Meropenem	Various metabolic enzymes	Central carbon metabolism, energy production
[118]	2022	*Yersinia pestis*, *Francisella tularensis*	Streptomycin, Gentamicin, Doxycycline	Various differentially expressed proteins	Fatty acid biosynthesis, TCA cycle, purine biosynthesis
[119]	2022	*Vibrio alginolyticus*	Serum resistance factors	Mannitol transporters, glycolytic enzymes, pyruvate cycle enzymes, cAMP/CRP	Metabolic pathways involving glycine, serine, threonine, fructose, mannose, and pyruvate, alongside central carbon metabolism and the biosynthesis of amino acids
[120]	2022	*Staphylococcus aureus* MRSA	Oxadiazolones	FabH, FphC, AdhE, FphE	Fatty acid biosynthesis pathway, redox and energy metabolism
[121]	2021	*Aeromonas hydrophila*	Enoxacin	Multidrug efflux transporters, DNA repair proteins, transcriptional regulators	DNA damage, SOS response, stress response and membrane transport
[122]	2021	*Klebsiella pneumoniae*	Cotrimoxazole, Amikacin	GarK, uxaC, exuT, hpaB, fhuA, KPN_01492, fumA, hisC, aroE	TCA cycle, alcohol metabolic process, folate biosynthesis
[123]	2020	*Escherichia coli*	β-lactams	A total of 1553 differentially expressed proteins	Purine metabolism, translation, transcription
[124]	2020	*Helicobacter pylori*	Daphnetin	Various membrane, repair and stress related proteins	Metabolism, membrane structure, nucleic acid and protein synthesis, ion binding, stress response
[125]	2019	*Staphylococcus xylosus*	Tylosin	A total of 155 differentially expressed proteins	Stress response, biosynthesis of amino acids, carbon metabolism, ABC transporters
[126]	2019	*Salmonella Typhimurium*, *Klebsiella pneumoniae*, *Staphylococcus aureus*	Various antibiotics	PrsA, YadC, FimA, RplB, AcrB, RpoB	Efflux, stress response, energy metabolism and redox processes, translation and transcription machinery
[127]	2018	*Edwardsiella piscicida*	Kanamycin	Various outer membrane proteins and type III secretion system related proteins and regulators	Biosynthesis of amino acids, 2-oxicarboloxylic acid metabolism, biosynthesis of secondary metabolites and metabolic pathways
[128]	2016	*Aeromonas hydrophila*	Chlortetracycline	Propanoate and fatty acid biosynthesis metabolism-related proteins	Biofilm formation, fatty-acid biosynthesis, propanoate metabolism

## Data Availability

No new data were created or analyzed in this study. Data sharing is not applicable to this article.
